# Spectra of Internal Friction in Polyethylene

**DOI:** 10.3390/polym14040675

**Published:** 2022-02-10

**Authors:** Viktor A. Lomovskoy, Svetlana A. Shatokhina, Anatoly E. Chalykh, Vladimir V. Matveev

**Affiliations:** Frumkin Institute of Physical Chemistry and Electrochemistry, Russian Academy of Sciences (IPCE RAS), Leninskiy Prospekt 31, 119071 Moscow, Russia; lomovskoy49@gmail.com (V.A.L.); matveev46@yandex.ru (V.V.M.)

**Keywords:** polyethylene, internal friction spectra, local dissipative processes, relaxation time, shear modulus defect, dissipation mechanisms

## Abstract

The study of spectra of internal friction $\lambda =f\left(T\right)$ and temperature dependencies of frequency of freely damped $\nu =f\left(T\right)$ oscillatory process excited in investigated samples of polyethylene with different degree of crystallinity in the temperature range from −150 ${}^{{}^{\circ}}$C to +150 ${}^{{}^{\circ}}$C. It is established that four local dissipative processes of different intensity shown in different temperature intervals are observed on the spectra $\lambda =f\left(T\right)$. These are μ, β, α, $\beta_{k}$ processes. The theoretical analysis of the relationship between the anomalous changes of the vibrational process frequency $\nu =f\left(T\right)$ and the shift modulus defect $\Delta G=f\left(T\right)$ and the internal friction mechanisms for each of the dissipative loss processes detected on the spectrum $\lambda =f\left(T\right)$ is carried out. The influence of supramolecular structures on local dissipative $\beta_{k}$ process in polyethylene is estimated.

## 1. Introduction

Recently, a large number of studies aimed at the study of rheological, strength and physical–mechanical properties of PE and polymer composite materials derived from it have been conducted [1,2,3,4]. At the same time, along with the study of fracture processes of these materials, the analysis of the phase state of the systems and their supramolecular structure, local dissipative processes (including relaxation processes) are also studied. It is established [5,6,7,8,9,10,11,12,13,14,15,16,17,18,19,20,21,22,23] that these processes are associated with the manifestation of the mobility of various structural elements. Therefore, the smallest structural changes lead to significant changes in the parameters of these local dissipative processes and characteristics of the whole system under study.

However, early investigations of relaxation processes in amorphous-crystalline polyethylene [24,25], carried out in the temperature range from −200 ${}^{{}^{\circ}}$C to +150 ${}^{{}^{\circ}}$C by dynamic methods at various frequencies of an oscillatory process excited in the system under study, have shown only a qualitative picture of distribution over temperature regions of local dissipative processes on the internal friction spectrum $\lambda =f\left(T\right)$ [3,24,25,26,27,28,29]. It was established [24] that three local dissipative processes of different intensity are observed on the spectra $\lambda =f\left(T\right)$. They are: β-relaxation process (temperature range ∼−150 ${}^{{}^{\circ}}$C ÷ −80 ${}^{{}^{\circ}}$C) related to mobility of groups $CH_{2}$ in macromolecule chains, α-relaxation process (temperature range from −40 ${}^{{}^{\circ}}$C to +40 ${}^{{}^{\circ}}$C) related to mobility of chain segments and $\beta_{k}$-process (temperature range from +40 ${}^{{}^{\circ}}$C to +100 ${}^{{}^{\circ}}$C). However, it is characteristic of the spectra obtained in the cited papers that they do not belong to fine structure relaxation spectra. The experimental points in them were obtained in large temperature intervals (10–20 K). Therefore, blurred maxima were observed [24,25] if so, please change to citation format, which did not reveal such a complex structure that was obtained in this work. In addition, the temperature dependencies of the frequency $\nu =f\left(T\right)$ of the freely damped oscillatory process and the theoretical analysis of the relationship between the anomalous change in the frequency $\nu =f\left(T\right)$ of the oscillatory process and the shear modulus defect $\Delta G=f\left(T\right)$ were obtained for the first time during the experiment.

The aim of the present work is more detailed investigation of local dissipative processes found on PE internal friction spectra, corresponding to these dissipative processes, anomalous phenomena in temperature dependencies of frequency of free damping oscillatory process, excited in PE samples of different crystallinity degree, with and without filler, monomodal and bimodal.

In order to achieve this goal, the following objectives were pursued:

1. Studies of spectra of internal friction $\lambda =f\left(T\right)$ in the temperature range from −150 ${}^{{}^{\circ}}$C to +150 ${}^{{}^{\circ}}$C in the mode of free damped torsion oscillations excited in PE samples;
2. Obtaining the temperature dependencies of the frequency $\nu =f\left(T\right)$ of a freely damped oscillatory process;
3. A theoretical analysis of the relationship between the anomalous change in the frequency $\nu =f\left(T\right)$ of the oscillatory process and the shear modulus defect $\Delta G=f\left(T\right)$ for each of the local dissipative process $\lambda =f\left(T\right)$ detected on the spectrum is carried out, and the magnitude and sign of the modulus defect ΔGx is calculated [30].
4. Identification of the internal friction mechanism for each local dissipative loss peak exhibited on the internal friction spectrum.

## 2. Experimental

### 2.1. Materials

Polyethylene pellets of the following grades were taken as samples for the study: HDPE 277-73 (Stavrolen, Budennovsk, Russia); BorSafe HE3490-IM (Borealis, Vienna, Austria); CRP100 Hostalen (Basell Polyolefins, Rotterdam, The Netherlands); Stavrolen PE4PP-25B (Stavrolen, Budennovsk, Russia). Carbon black (carbon black) 2 ± 0.5 wt% was used as a light stabilizer. The main characteristics of the studied PE are given in Table 1.

HDPE 277-73 and Stavrolen PE4PPP-25B were synthesized by gas-phase low-pressure ethylene polymerization on complex metal-bound catalysts (using Unipol technology). Carbide technology, referred to as Unipol, is the most popular technology in the world [31]). HE3490-IM (Borsafe) is produced by advanced Borstar technology which consists of two reactors in series, a circulating loop reactor and a low pressure gas phase reactor. CRP 100 Hostalen has been produced using the Hostalen process—suspension polymerization in stirred reactors [32].

Hostalen suspension polymerization is carried out in two reactors operating in parallel or in series. Switching from one reactor to cascade made it possible to produce high quality single and bimodal polyethylene with any molecular weight distribution using the same catalyst. Polymerization is carried out in a solvent, such as n-hexane, over a highly reactive Ziegler catalyst. At the end of the process, it is not necessary to deactivate the catalyst and extract it from the polymer because the concentration of residual catalyst in the polymer is very low. To obtain a unimodal product, the catalyst, solvent, monomer and hydrogen are introduced into reactors 1, 2 where polymerization takes place. To obtain bimodal grades, the catalyst is introduced only in the first reactor 1; the second stage of polymerization proceeds under conditions different from those of the first reactor. In the second reactor 2 ethylene, butene and additional amount of solvent are introduced. Process conditions are continuously controlled, which provides very high quality polyethylene [32].

### 2.2. Methods

**Figure 1:**
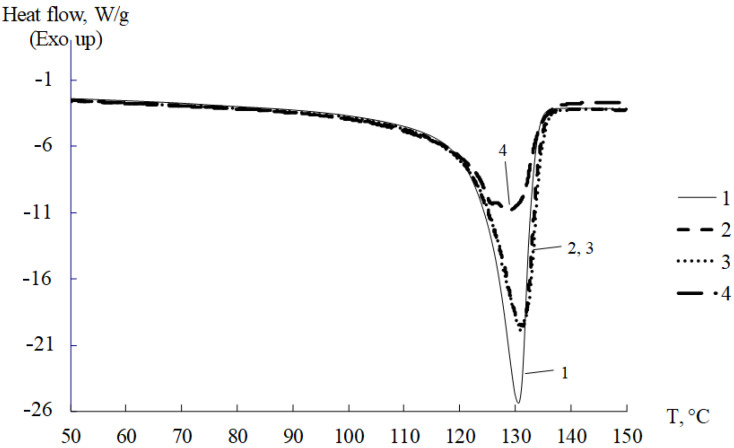
DSC thermograms for PE of different grades: 1—PEVP 277-73; 2—HE3490-IM; 3—CRP 100 Hostalen; 4—Stavrolen PE4PP-25B.

The degree of crystallinity of the polyethylenes under study was determined by differential scanning calorimetry on a DSC Q100 unit of Intertech Corporation (USA) at a rate of 5 °C/min at an argon current of 50 mL/min. Typical DSC curves are shown in Figure 1. Comparable results were obtained by X-ray analysis of the degree of crystallinity [33,34], so we limited ourselves to the results obtained by the DSC method.

The observed peaks in the thermograms correspond to endothermic melting processes of the PE crystalline phase and are in the range. The degree of crystallinity of α PE samples was determined using the traditional relation [35].

(1) $$\alpha =\frac{\Delta H_{m}}{\Delta H_{0}}\cdot 100\%\;$$

where $\Delta H_{m}$ is enthalpy of melting of PE samples; $\Delta H_{0}$—theoretical value of polymer melting enthalpy with 100% degree of crystallinity. $\Delta H_{0}$ = 293 kJ/kg [35,36,37].

**Figure 2:**
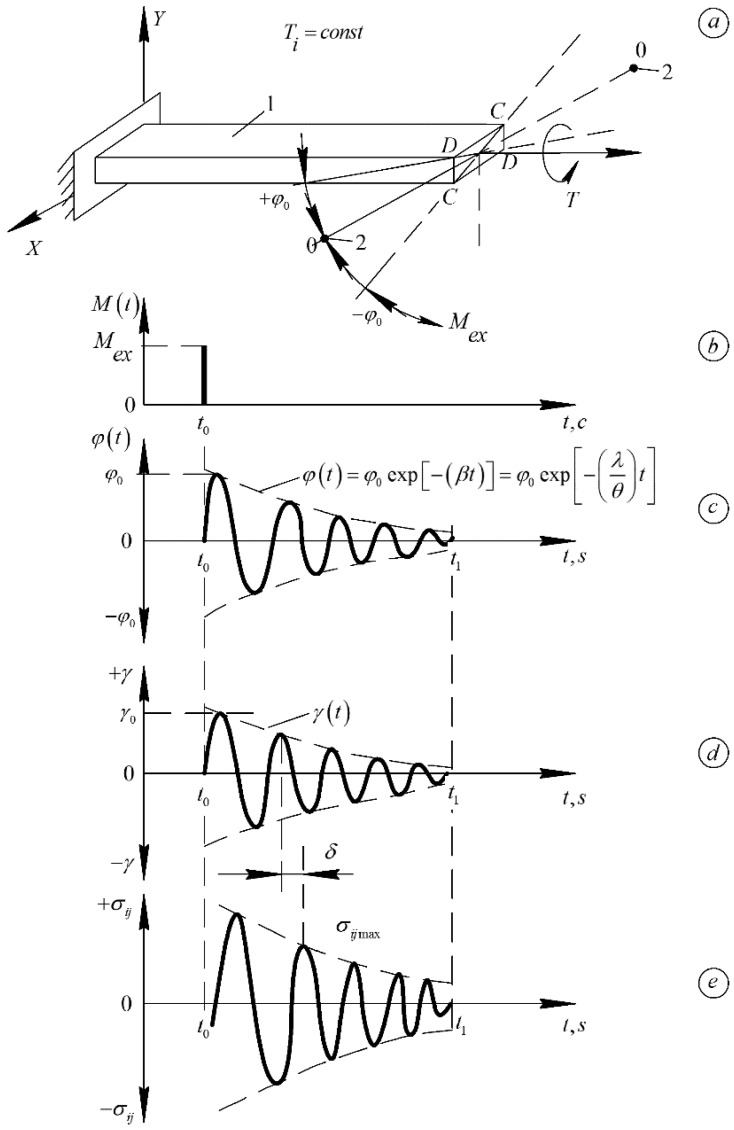
Diagrams of freely damped oscillatory process excited in the studied sample; (**a**) in isothermal mode T=const; (**b**) by pulse action. Sweep of the time dependence of the twist angle $\varphi \left(t\right)$ relative to the longitudinal axis *Z* of the specimen (**c**). The deformation of the sample—$\gamma \left(t\right)$ (**d**) and the corresponding shear stresses $\sigma_{ij}$ occurring in the sample (**e**). β—damping coefficient of the oscillatory process; θ—period of the vibration process. All other designations are defined below in the text of the article.

PE samples in the form of plates of rectangular cross-section with thickness of 1 mm, length of 65 mm, width of 5 mm were obtained by pressing in a closed-type mold at temperature *T* = 180 ${}^{{}^{\circ}}$C. The spectra of internal friction $\lambda =f\left(T\right)$ and temperature dependencies of the frequency $\nu =f\left(T\right)$ of free damping oscillations excited in PE samples were obtained on a horizontal torsion pendulum [38]. Figure 2 shows schematically the methodology for obtaining these experimental results.

The technique of obtaining spectra of internal friction $\lambda =f\left(T\right)$ and temperature dependencies of frequency $\nu =f\left(T\right)$ of free damped oscillations excited in PE samples consists in the following. Samples of investigated polymers were fixed in clamps of horizontal pendulum (Figure 2) and subjected to deforming influence of torsion after submission of external pulse influence (Figure 2b) on vibrating system of the device. The mode of external deforming influence is described by the relation:

(2) $$M_{ex}=M\left(t\right)\cdot \delta \left(t\right)=\left\{\begin{array}{c} 0\;\mathrm{as}\;t<t_{0}\\ M_{ex}\;\mathrm{as}\;t=t_{0}\\ 0\;\mathrm{as}\;t>t_{0} \end{array}\right.$$

where δ(t) is the Dirac delta function.

As a result of impulse action, the investigated sample makes damped torsional oscillations in the $t<t_{0}<t_{1}$ range near the position of equilibrium $\varphi \left(t\right)=0$ (Figure 2c). At the same time, shear deformations $\gamma \left(t\right)$, which are related to the logarithmic decrement, appeared in the sample by the following relation:

(3) $$\gamma \left(t\right)=\gamma_{0}\exp{\left[-\left(\frac{\lambda }{\pi }\right)\right]}^{t}$$

where λ is the logarithmic decrement of the oscillatory process excited in the studied sample, determined for each temperature by the relation:

(4) $$\lambda =\frac{1}{N-1}\ln\frac{\varphi_{1}}{\varphi_{N}}$$

where *N* is a number of periods of oscillatory process from the 1-st, where the amplitude is equal $\varphi_{1}$ to the *N*-th, where the amplitude is equal to $\varphi_{N}$. It is established, that periodic oscillatory process excited in investigated samples, in the general case is quasiharmonic, but in the first approximation, the logarithmic decrement can be assumed as constant on all time sites of these isothermal process sweeps.

Since the controlled parameter of the transient oscillatory process from the excited nonequilibrium state to the equilibrium state is the logarithmic decrement λ, the set of values λ for each temperature in the temperature interval under study represents the spectrum of internal friction $\lambda =f\left(T\right)$. Besides, each transition process to the equilibrium state is characterized for each temperature by its frequency of the damped oscillatory process. This allows us to obtain in the required temperature range of research the temperature dependence of the frequency of $\nu =f\left(T\right)$ freely damped oscillatory process excited in the investigated sample. The frequency ν of the oscillatory process was determined automatically by the sweep of the oscillatory process for each temperature.

The temperature range of the experiment was from −150 ${}^{{}^{\circ}}$C to +150 ${}^{{}^{\circ}}$C. The heating rate of the samples under study in the thermal cryocamera was 2${}^{{}^{\circ}}$ per minute.

**Figure 3:**
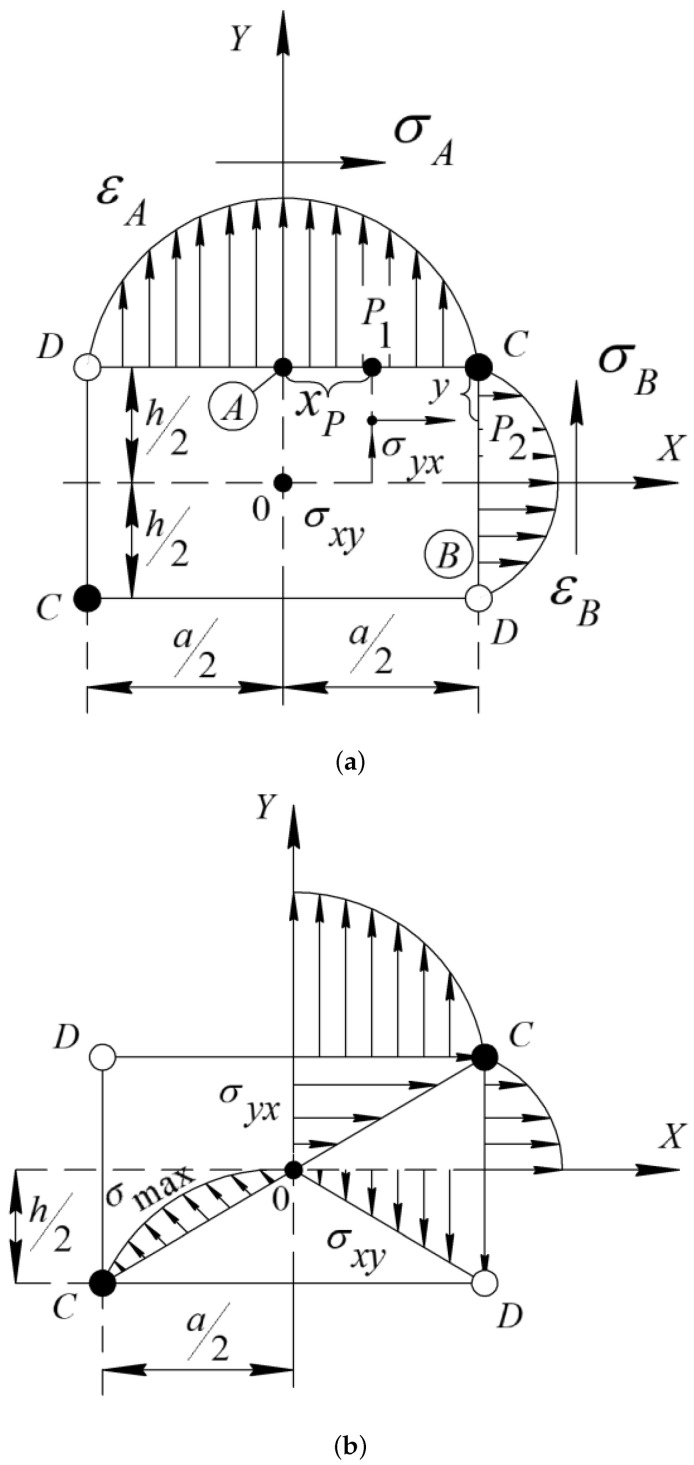
Diagrams of stress distribution over the cross section of the investigated PE sample at free damping torsional oscillations, excited in this sample: (**a**) along the faces; and (**b**) along the diagonals.

The stresses in the cross-section of the sample are distributed as follows (Figure 3a,b): on the wide face $\sigma =\sigma_{yx_{\max}}$, on the narrow face $\sigma =\sigma_{xy_{\max}}$. Thus, the greatest stress will be on the surface of the face that is closer to the axis of the beam. The maximum value of stress on the diagonal is smaller than the stresses on the faces of the sample. This range of stress variation is in the interval significantly lower than the proportional limit of the materials investigated in this work $\sigma_{pr}$ = 5 N·mm${}^{-2}$. The obtained values of maximum stresses are significantly lower than the proportional limit $\sigma_{pr}$ and elastic limit, i.e., $\sigma_{el}$ the relations $\sigma_{ii}<\sigma_{pr}$ and $\varepsilon_{ii}<\varepsilon_{pr}$ or for tangential stresses $\sigma_{ij}<\sigma_{pr}$ and $\varepsilon_{ij}<\varepsilon_{pr}$, and amplitude-independent internal friction [4] are satisfied.

Calculation of the error of these measurements on the maximum values of the stresses $\sigma_{ij}$ occurring on the surfaces of the faces and cross-section of the studied samples and the corresponding values of shear deformation $\gamma_{ij}$ does not exceed 2%.

The structural and morphological characteristics of polyolefins were studied by transmission electron microscopy. The outer surface of the samples was used as an object of study, which was subjected to etching in high-frequency oxygen plasma to reveal their supramolecular structure. Oxygen pressure in the etching area was 0.03 mm Hg, electron energy—2–3 eV, etching time—not more than 15 min, generator power—100 W, frequency—10 MHz. The morphology of etched surfaces was examined by one-step carbon-platinum replicas using transmission electron microscope Philips EM301 (Tokyo, Japan) at accelerating voltage of 80 keV.

## 3. Results and Discussion

### 3.1. Experimental Results

**Figure 4:**
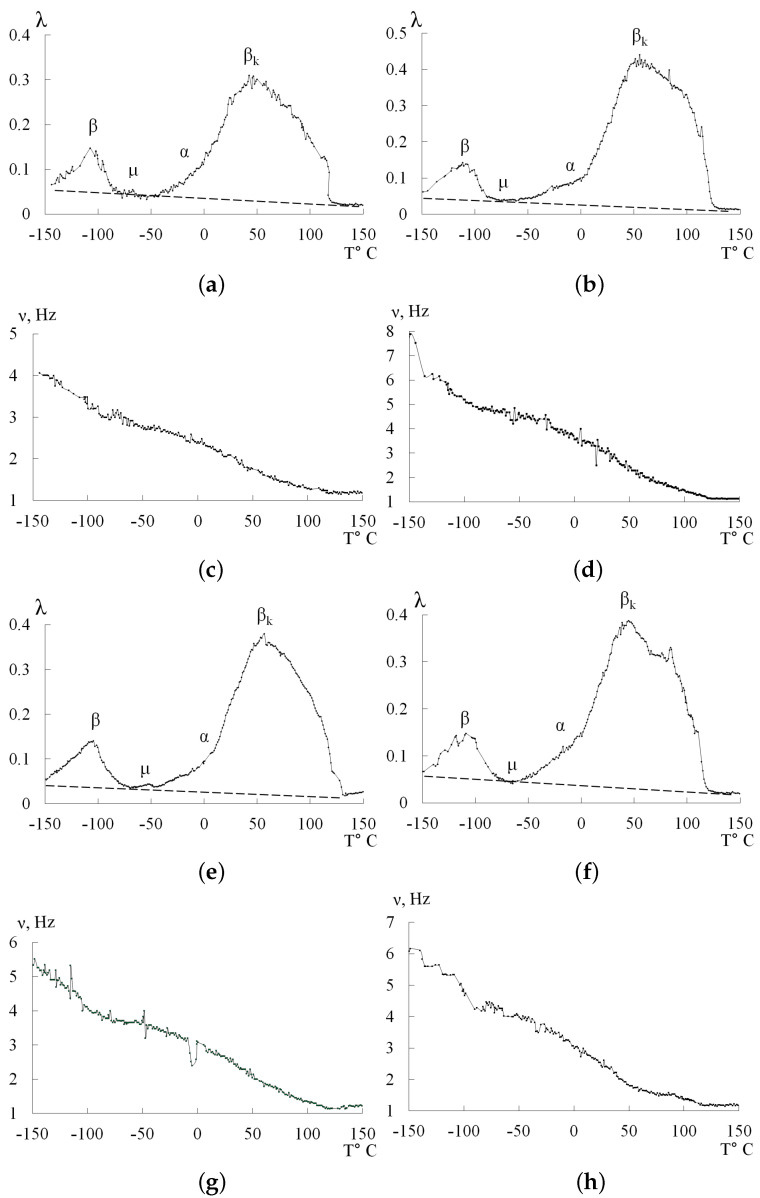
Internal friction $\lambda =f\left(T\right)$ and temperature-dependent frequency spectrum $\nu =f\left(T\right)$ for HDPE 277-73 (**a**,**c**); HE3490-IM (**b**,**d**); CRP100 Hostalen (**e**,**g**); Stavrolen PE4PP-25B (**f**,**h**).

Figure 4 shows obtained spectra of internal friction $\lambda =f\left(T\right)$ for PE of different grades and temperature dependencies of frequency $\nu =f\left(T\right)$ of free damped torsion oscillations excited in investigated samples in the temperature range from −150 ${}^{{}^{\circ}}$C to +150 ${}^{{}^{\circ}}$C.

**Figure 5:**
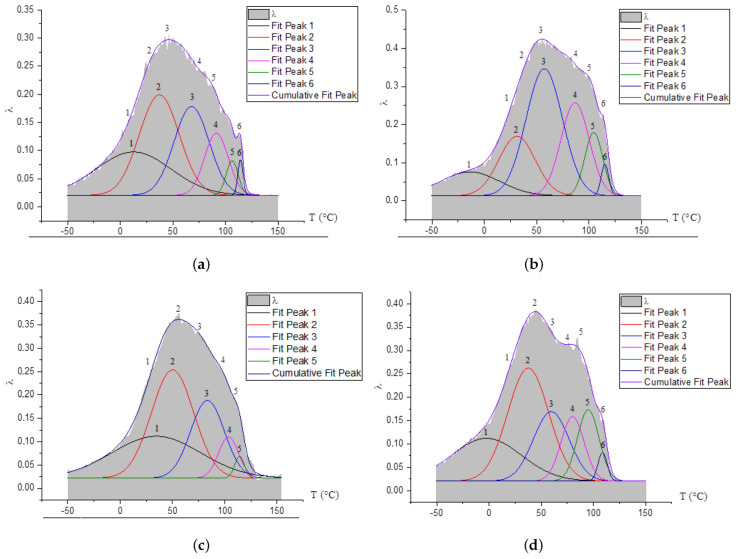
Decomposition of $\beta_{k}$-peak dissipative losses by mathematical method using Gaussian normal distribution for HDPE 277-73 (**a**); HE3490-IM (**b**); CRP100 Hostalen (**c**); and Stavrolen PE4PPP-25B (**d**).

As a result of mathematical processing (Figure 5), the obtained experimental peaks of dissipative losses ($\beta_{k}$-process) were decomposed using the Gaussian normal distribution into several local subscripts of dissipative losses ($\beta_{k_{i}}$). The number *i* of these subpeaks does not depend on the degree of crystallinity, but is related to configuration factors determined by polyethylene synthesis and amounts to 5–6 subpeaks. Each *i*-subspike characterizes the mobility of a specific structural-kinetic unit within an amorphous interfacial layer. As a result of mathematical processing of the experimental peak, it was found that these local subpeaks appear at different temperatures, which are included in the general temperature interval of the whole $\beta_{k}$-process and characterize different conformational positions of the pass-through chains in the amorphous interfacial PE layer.

**Figure 6:**
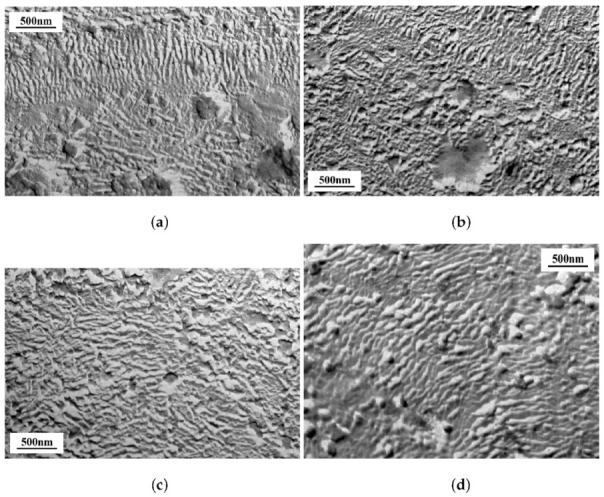
Electron microphotographs of HDPE: HDPE 277-73 (**a**); HE3490-IM (**b**); CRP100 Hostalen (**c**); and Stavrolen PE4PPP-25B (**d**).

The morphological pictures of PE supramolecular organization obtained by us, presented in Figure 6, confirm the above observation that «the smallest structural changes in polyolefins lead to significant changes in the parameters of these local dissipative processes and characteristics of the whole system under study». It can be seen that in all PE samples investigated, there are at least two crystallizing structural elements—lamellae and fibrillar crystals forming different secondary macroformations—spherolites, macrofibrils, and ribbons. Each of these formations corresponds to different morphologies of amorphous phases, which include transitional layers of regular and irregular chains; remote irregular loops and passing chains; fibril-like regions consisting of extremely straightened chains, binding fibrils and crystallites.

Two temperature regions of the most intense local dissipative processes (β and $\beta_{k}$) are observed on the spectra $\lambda =f\left(T\right)$ in the temperature range from −150 ${}^{{}^{\circ}}$C to +150 ${}^{{}^{\circ}}$C for PE. The temperature regions of these processes partially coincide with the data published earlier [39,40,41,42,43,44,45,46]. In addition, two temperature regions with very weak intensity of local dissipative processes are identified on the spectra $\lambda =f\left(T\right)$ and temperature dependencies of the frequency $\nu =f\left(T\right)$ of freely damped oscillatory process. We assume that it is the α dissipative process practically completely absorbed, superposition of the low-temperature branch $\beta_{k}$-process and μ-process, observed in the region of temperatures ∼−70 ${}^{{}^{\circ}}$C.

The most complex and the most intensive dissipative process on spectra $\lambda =f\left(T\right)$ for all investigated PE systems is $\beta_{k}$-process. This process is determined by the mobility of structural elements of the amorphous phase adjacent to the boundaries of various crystalline formations in the amorphous-crystalline PE structure and can be decomposed into several dissipative processes superimposed on each other by temperature, which confirms the data of studies obtained by other methods [41] (Figure 5).

The second intensive peak of dissipative losses β, located on a spectrum $\lambda =f\left(T\right)$ in the temperature interval from −140 ${}^{{}^{\circ}}$C to −90 ${}^{{}^{\circ}}$C is also, as well as $\beta_{k}$-process, is complex process. Complexity of this process is shown in occurrence of sharp, local on temperature, bursts of intensity of dissipative losses, observed both on low-, and on high-temperature branches of β-process. Following Bartenev, the structural mechanism of this dissipative process is determined by the mobility of structural-kinetic elements $CH_{2}$ [24].

The next local dissipative process has much less intensity $\lambda_{\max}$ (Figure 4) and is observed in the temperature range from −90 ${}^{{}^{\circ}}$C to −60 ${}^{{}^{\circ}}$C. This is a μ dissipative loss process.

The fourth dissipative process is located in an intermediate temperature region between μ both $\beta_{k}$ processes, has very low intensity on the spectrum $\lambda =f\left(T\right)$ (Figure 4) and appears as an increasing background of dissipative losses on the spectrum. It is a relaxation process α related to segmental mobility of macromolecule structural elements in the amorphous PE subsystem. This process is almost completely absorbed by the low-temperature branch $\beta_{k}$-the relaxation process.

Our data on temperatures of maxima clearly observed on spectra of internal friction and values of activation energy calculated from experimental results are in agreement with the results obtained earlier in a number of studies conducted by methods of DSC, DMA and RTL [43,44,45,46]. On the spectra of internal friction obtained on a horizontal torsion pendulum, these maxima are observed more clearly than, for example, on DSC. In addition, the entire spectrum consists of a large number of peaks, each of which has its own values of temperatures, intensities, frequencies, and calculated values of the activation energy and relaxation time. The structural nature of the observed peaks and their temperature positions are still under debate [43,44,45,46], which testifies to the relevance of this topic at present.

All dissipative processes detected on spectra of internal friction $\lambda =f\left(T\right)$ are superimposed on internal friction background (dot-and-dash line, Figure 4a,b,e,f). Distinctive feature of obtained experimental results is decrease of intensity of dissipative losses background (internal friction background) at increase of investigation temperature. In previous studies [15,20,21,22,23], on the contrary, there is a sharp increase of internal friction background at temperatures above the $\beta_{k}$-process temperature.

Thus, the obtained spectra have significant differences from the spectra obtained earlier [24,41]. These differences are as follows:

1. Low intensity of α-process relative to the intensity of β- and $\beta_{k}$-processes;
2. Significant complexity of the $\beta_{k}$-process on both the low temperature side $T<T_{\beta_{k}}$ and the higher temperature side $T>T_{\beta_{k}}$;
3. The background of internal friction, on which the dissipative loss peaks are superimposed, decreases when the temperature rises from −150 ${}^{{}^{\circ}}$C to +150 ${}^{{}^{\circ}}$C.

It should be noted that the temperature position (temperature of maximum $T_{\beta_{\max}}$ and intensity $\lambda_{\beta_{\max}}$ losses) for the β-process on a spectrum $\lambda =f\left(T\right)$ for all investigated PE grades (Figure 3) practically does not depend on degree of crystallinity of polyethylene. The structural mechanism of this dissipative process is determined by the mobility of $CH_{2}$-structural-kinetic elements in the amorphous PE phase, which is not part of crystalline formations.

**Figure 7:**
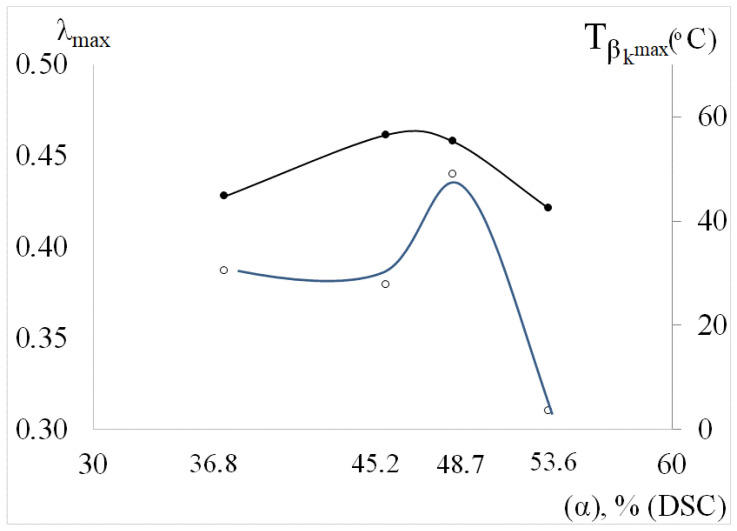
Dependence plot $T_{\beta_{k\max}}$ (along the auxiliary axis) and the vibrational $\lambda_{\beta_{k\max}}$ process on the degree of crystallinity of the polymer.

For the $\beta_{k}$ dissipative process, the temperature position of the maximum $T_{\beta_{k\max}}$, the intensity of losses $\lambda_{\beta_{k\max}}$, the frequency of the oscillatory process $\nu_{\beta_{k\max}}$ at the temperature of maximum loss $T_{\beta_{k\max}}$, as well as the temperature interval of the dissipative loss $\Delta T_{\beta_{k\max}}$ peak on the spectrum $\lambda =f\left(T\right)$ are functions of the degree of crystallinity α, % of PE (Figure 7).

Simultaneously with the measurement of the logarithmic decrement λ of the damped oscillatory process, we studied the temperature dependence of the frequency of the oscillatory process excited in the studied PE samples (Figure 4). The temperature dependencies of the frequency $\nu =f\left(T\right)$ of free damping torsion oscillations excited in the studied PE samples indicate the temperature regions of anomalous frequency changes in those intervals in which dissipative loss peaks are observed on the spectra $\lambda =f\left(T\right)$.

For the β- and $\beta_{k}$-dissipative processes on the spectrum $\lambda =f\left(T\right)$ (Figure 4) in the temperature intervals corresponding to these processes on the temperature dependence of the frequency $\nu =f\left(T\right)$ of the oscillatory process is observed local by temperature and relatively sharp decline in the frequency ν of oscillatory process.

It should be noted that in the temperature region of the manifestation μ-dissipative process on the temperature dependence $\nu =f\left(T\right)$ (in contrast to β- and $\beta_{k}$-dissipative processes) there is an increase in the frequency of the oscillatory process (Figure 5).

In the temperature region of manifestation α-dissipative process on the dependence $\nu =f\left(T\right)$, no anomalousness is found.

The obtained experimental temperature dependencies of the frequency $\nu =f\left(T\right)$ of free damped torsion oscillations excited in the examined PE samples during their theoretical treatment allow determining the following physico-chemical and physico-mechanical characteristics of the polymer:

1. The magnitude of the shear modulus defect (i.e., the anomalous shear modulus decrease that significantly differs from the theoretical temperature dependence of the modulus decrease with temperature increase) for each of the local dissipative processes detected on the internal friction spectrum;
2. Determine the mechanism of internal friction of a given local dissipative process located in the same temperature region of research by the magnitude of shear modulus defect ΔG, or more precisely by the sign of magnitude;
3. Calculate the real temperature decrease of the shear modulus $G=f\left(T\right)$ for all investigated PEs over the whole temperature interval taking into account the influence of local shear modulus defects observed in different temperature intervals on the value of this modulus.

### 3.2. Theoretical Analysis of Experimental Results

As follows from the experimental spectra of internal friction as well β- as $\beta_{k}$-local dissipative processes appear to be very complex, consisting of a set of several processes manifested in the same temperature intervals.

The β-relaxation process in PE is characterized by the presence of sharp, but narrow in the temperature range of manifestation, insignificant intensity bursts of dissipative losses, both on low-molecular and high-molecular branches of the losses peak (Figure 4a,b,e,f). The nature of this phenomenon is unclear; however, it should be noted that similar effects have been observed in the internal friction spectra of other vinyl polymers (in particular, in polyvinyl alcohol) [47,48]. In these polymers, this effect is probably due to the presence of various forms of molecular water in the structure. According to the data given in [49], PE can absorb up to ∼0.04% of water in an aqueous medium. Therefore, in principle, dissipative effects associated with the presence of both molecular and droplet water in the PE structure are possible on the internal friction spectra. On the other hand, the manifestation of dissipative phenomena associated with the transformations of molecular water from amorphous $I_{L}$ and cubic structures $I_{C}$ to hexagonal structures $I_{h}$ [47] should have simply been absorbed by the more intense β-relaxation process in PE. This is due to low-intensity μ-processes associated with the local dissipative mobility of different water forms in the temperature range from −170 ${}^{{}^{\circ}}$C to −140 ${}^{{}^{\circ}}$C. The only possible process associated with the local dissipation caused by the presence of water in the PE structure is the μ-process observed in the temperature range from −90 ${}^{{}^{\circ}}$C to −70 ${}^{{}^{\circ}}$C. The crystalline hexagonal form of water $I_{h}$ is present in the PE structure in this interval and its manifestation on the internal friction spectrum as a low-intensity dissipative loss peak is accompanied by a negative value of the shear modulus defect ΔG<0, which is also observed on the temperature dependence $\nu =f\left(T\right)$ for PE (Figure 4c,d,g,h). The negative value ΔG indicates a phase rather than relaxation mechanism of dissipative losses [47]. All other sharp and intense bursts of dissipative losses on the spectrum $\lambda =f\left(T\right)$ in the β-process region of relaxation, apparently, can be related to different structural organization of the amorphous phase in the amorphous–crystalline PE structure.

The main attention in this paper is paid to the $\beta_{k}$ dissipative process. According to the phenomenological provisions of the internal friction theory the peak of dissipative losses on the spectrum $\lambda =f\left(T\right)$ can be described by the differential equation of the standard linear body model:

(5) $$\frac{d\sigma }{dt}+\frac{G_{1}}{\eta }\sigma =\left[\left(G_{1}+G_{y}\right)i\omega +\frac{G_{1}G_{y}}{\eta }\right]\gamma_{0}\exp i\omega t$$

where σ—stresses arising in the system under study; $G_{1}$ and $G_{y}$—shear modulus of the subsystem causing the appearance of a loss peak on the spectrum $\lambda =f\left(T\right)$ and shear modulus of the aggregate shaping subsystem causing the appearance of dissipative loss background on the spectrum $\lambda =f\left(T\right)$, respectively; η—viscosity of the polymer subsystem under study causing the appearance of a loss peak; ω—angular frequencyof the oscillatory process caused in the system under study.

The solution of this differential equation for the damped oscillatory process (taking into account that the shear modulus $G_{1}$ takes a complex form) leads to a relation of the form:

(6) $$\lambda_{i}=2\lambda_{\beta_{k\max}}\frac{\omega \tau }{1+{\left(\omega \tau \right)}^{2}}$$

where $\lambda_{i}$ and $\lambda_{\beta_{k\max}}$ are the current and maximum values of the logarithmic coefficient of the damped oscillatory process for the $\beta_{k}$ dissipative process; $\tau \equiv \tau_{\beta_{k}}=\frac{\eta }{G_{1}}$ is relaxation time of the subsystem, causing the appearance of the dissipative loss peak on the spectrum $\lambda =f\left(T\right)$.

It follows from relation (6) that the current change in temperature $\lambda_{i}$ reaches its maximum at the peak of losses $\left(\lambda_{i}=\lambda_{\beta_{k\max}}\right)$ when the condition is fulfilled:

(7) $$\omega \tau_{i}=1$$

where the relaxation time $\tau_{i}$ is a function of temperature $T_{i}$, i.e.,

(8) $$\tau_{i}=\tau_{0}\exp\frac{U_{\beta_{k\max}}}{RT_{i}}$$

where $U_{\beta_{k\max}}$ is activation energy of the dissipative process; $\tau_{0}\approx 1.6\times 10^{-13}$ s is theoretical value of the pre-exponential coefficient characterizing the oscillatory process of the relaxing particle at the bottom of the potential well. The frequency of the oscillatory process ν (determined experimentally from the dependence $\nu =f\left(T\right)$) is related to the circular frequency ω by the relation: ω=2πν. This allows us to determine the relaxation time $\tau =\tau_{\max}$ in the local dissipative loss peak $\lambda_{\max}$ according to the frequency $\nu =\nu_{\max}$ value corresponding to this value in the temperature dependence by $\nu =f\left(T\right)$ the relation of the form:

(9) $$\omega_{\max}\tau_{\max}=2\pi \nu_{\max}\tau_{\max}=2\pi \nu_{\left(T=T_{\max}\right)}\tau_{\left(T=T_{\max}\right)}=1\Rightarrow \tau_{\left(T=T_{\max}\right)}=\frac{1}{2\pi \nu_{\left(T=T_{\max}\right)}}$$

The activation energy of this process is determined from the Arrhenius dependence of relaxation time τ on temperature (relation 8) taking into account (9) in the form:

(10) $$U_{\beta_{k\max}}=RT_{\beta_{k\max}}\ln\frac{\tau_{\beta_{k\max}}}{\tau_{0}}$$

Obtained, according to the above formulas, physical–mechanical and physical–chemical characteristics for the dissipative loss process $\beta_{k}$ are presented in the table (Table 2).

In order to determine the mechanism of internal friction, for each of the dissipative processes detected on the spectrum $\lambda =f\left(T\right)$ of internal friction, it is necessary to calculate the shear modulus defect, which is done on the basis of the experimentally obtained temperature dependence of the frequency of the vibrational process excited in the studied PE samples. Each local dissipative process observed on the spectrum $\lambda =f\left(T\right)$ is characterized by its temperature interval, the magnitude and sign of the modulus defect $\Delta G_{i}$. The magnitude of the modulus defect $\Delta G_{i}$ is determined from the relation that takes into account the relationship between the change in the frequency of the oscillatory process excited in the studied sample and the change in the shear modulus of the material of this sample [50]. Taking into account the fact that in the used device, the investigated sample is an integral part of the vibrational system, the torsional vibrations excited in the investigated polymer can be considered as the modes of natural vibrations, where only the first mode is taken for the damped process [51]. In this case, the relationship between the dynamic shear modulus *G* and the frequency ν of the oscillatory process is defined as:

(11) ν=14G·IalIs+2Il1122

where *G* is shear modulus of the material of the investigated sample; $I_{a}$ is polar moment of inertia of the cross-section relative to the longitudinal (polar) axis of the investigated sample; $I_{s}$ is polar moment of the sample; *I* is polar moment of inertia of additional pole tips of the torsion oscillation excitation system, whose axis coincides with the longitudinal axis of the sample; *l* is length of the investigated sample. If in the first approximation, the temperature changes of moments of inertia of the vibrating system of the device can be neglected, then the relation (11) can be represented in the form:

(12) $$G\approx k_{1}\nu^{2}$$

**Figure 8:**
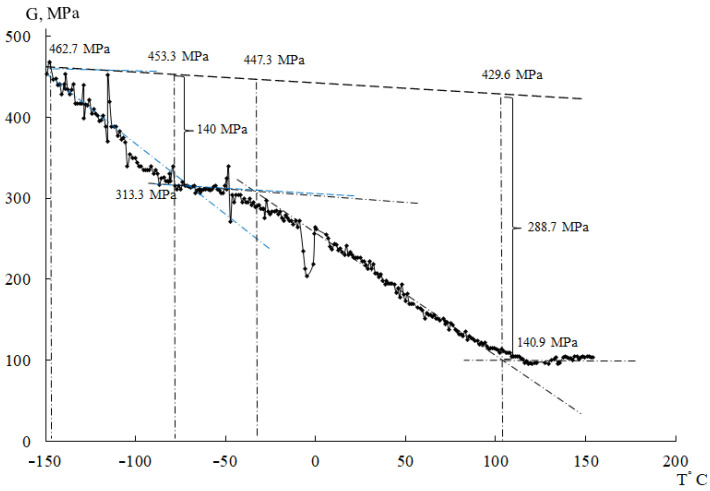
Temperature dependence of PE shear modulus—theoretical dependence—straight line 1 and curve 2 calculated with allowance for modulus defects for β and $\beta_{k}$ relaxation processes for CRP 100 Hostalen.

However, it was found experimentally that the shear modulus *G* is a function of temperature, i.e., $G=f\left(T\right)\equiv G\left(T\right)$. The theoretical dependence $G=f\left(T\right)$ when the temperature increases by every 100 degrees corresponds to a linear decrease in the modulus *G* by 2÷4% [52] (Figure 8, dot-and-dash line).

In this case, the frequency ν of the oscillatory process will also depend on the temperature, i.e., $\nu =f\left(T\right)\equiv \nu \left(T\right)$ and will change in proportion to the temperature change of the shear modulus $G\left(T\right)$, i.e.,

(13) $$G\left(T\right)k_{2}\equiv \nu^{2}\left(T\right)k_{1}$$

where $k_{1}$ and $k_{2}$ are constant coefficients.

Since $k_{1}=const$ and $k_{2}=const$ relation (13) can be represented in dimensionless form, i.e.,

(14) $$\frac{G_{i}\left(T_{i}\right)k_{2}}{G_{0}\left(T_{0}\right)k_{2}}=\frac{\nu_{i}^{2}\left(T_{i}\right)k_{1}}{\nu_{0}^{2}\left(T_{0}\right)k_{1}}\Rightarrow \Delta G\left(T\right)\approx \Delta \nu^{2}\left(T\right)$$

where $G_{i}\left(T_{i}\right)$ and $\nu_{i}^{2}\left(T_{i}\right)$ are the current temperature $T_{i}$ value of the shear modulus $G_{i}$ and frequency squared $\nu_{i}^{2}$; $G_{0}\left(T_{0}\right)$ and $\nu_{0}^{2}\left(T_{0}\right)$ are the initial value at the selected temperature $T_{0}$ of the shear modulus $G_{0}$ and frequency squared $\nu_{0}^{2}$.

Thus, the temperature change of the frequency $\nu \left(T\right)$ of an oscillatory process excited in the system under study allows us to determine the temperature change of the shear modulus $G\left(T\right)$ of the material from which the sample under study is made. However, experimental data of the $\nu =f\left(T\right)$ dependence study show that in certain temperature intervals, where local dissipative processes in the form of loss peaks are observed on the spectra $\lambda =f\left(T\right)$ of internal friction, an anomalous change in the temperature dependence of the frequency ν, and, consequently, shear modulus *G* caused by significant deviations from proportional theoretical temperature dependence $G=f\left(T\right)$ or $\nu =f\left(T\right)$. To describe this anomaly, we introduce the concept of shear modulus defect or frequency defect. The modulus defect is defined as a dimensionless quantity in the form:

(15) $$\Delta G\left(T\right)=\frac{G_{0}\left(T_{0}\right)-G_{i}\left(T_{i}\right)}{G_{0}\left(T_{0}\right)}=\frac{\nu_{0}^{2}\left(T_{0}\right)-\nu_{i}^{2}\left(T_{i}\right)}{\nu_{0}^{2}\left(T_{0}\right)}$$

Each local dissipative process detected on the spectrum $\lambda =f\left(T\right)$ of internal friction in different temperature intervals corresponds to a certain anomaly in the temperature dependence of the oscillation frequency $\nu =f\left(T\right)$, and hence, the shear modulus $G=f\left(T\right)$, which leads to a local temperature region of inelasticity of the studied system. In the general case, for each *i* of the dissipative loss peaks detected on the spectrum $\lambda =f\left(T\right)$ of internal friction there will be observed its own shear modulus defect $\Delta G_{i}\left(T\right)$ on the temperature dependence of the frequency $\nu =f\left(T\right)$ of oscillations. If we take into account that the shear modulus *G* of the whole investigated system is formed by addition of moduli $G_{i}$ of all *n*-structural-kinetic subsystems forming this system, i.e.,

(16) $$G_{i}=\sum_{i=1}^{n}G_{i}$$

then the temperature dependence of the shear modulus of the whole system will be defined as

(17) $$G\left(T\right)=\sum_{i=1}^{n}G_{i}\left(T\right)\;\pm \;\sum_{i=1}^{n}\Delta G_{i}\left(T\right)$$

Shear modulus defect can have positive value for dissipative processes of relaxation nature and negative value for dissipative processes of non-relaxation nature. The calculation of the magnitude and sign of shear modulus defects for all investigated systems by relation (15) is given in Table 3.

Calculations of temperature changes of the shear modulus $G\left(T\right)$ based on the temperature dependence of the frequency $\nu =f\left(T\right)$ of oscillatory process and the relative magnitude of shear modulus defects $\Delta G_{\beta }$ and $\Delta G_{\beta_{k}}$ for β, $\beta_{k}$ relaxation processes (on the example of CRP 100 Hostalen, Figure 8) allow quantitative determination of the real change in the strength characteristics of the studied materials, taking into account local temperature changes in the shear modulus caused by local dissipative losses introduced by each local dissipative process and manifested in the spectrum $\lambda =f\left(T\right)$.

### 3.3. Determination of the Phenomenological Mechanism of the Local Dissipative Process Detected on the Internal Friction Spectrum by the Sign of the Modulus Defect of This Process

**Figure 9:**
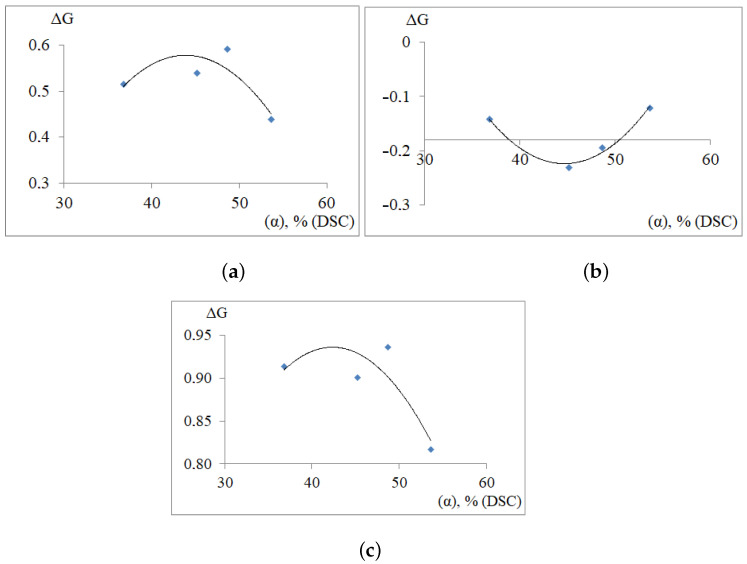
Dependence of the modulus defect on the degree of crystallinity $\Delta G=f\left(\alpha ,\%\right)$ for β (**a**); μ (**b**); $\beta_{k}$ (**c**) dissipative processes in PE.

Calculation of modulus defects ΔG (by relation 15) for investigated PE has shown that both β, $\beta_{k}$ processes have a relaxation mechanism of internal friction and the μ process has a non-relaxation mechanism (Table 3). Figure 9a–c show the dependencies of the modulus defect for PE on the degree of crystallinity $\Delta G=f\left(\alpha ,\%\right)$ for the processes β, μ and $\beta_{k}$, respectively.

The analysis of the obtained dependencies shows the generality of the change in the modulus defect in the α=45%. This qualitative generality is determined by the fact that, independently of α,% for all dissipative processes detected on spectra (β, μ and $\beta_{k}$) there is an anomaly of the extreme form, which is expressed as a certain peak on the dependence $\Delta G=f\left(\alpha ,\%\right)$. It seems that at this value of the crystallinity degree in PE structure such transformations take place that concern all dissipative processes. In this case, the maximum decrease in the shear modulus of the entire PE system (regardless of the grade) will be observed for a system with a crystallinity degree equal to α=45%.

## 4. Conclusions

On spectra of internal friction three (the fourth-α-process of relaxation is almost completely absorbed by low-temperature branch of $\beta_{k}$-process of relaxation) local dissipative processes of different intensity, shown at different temperatures are revealed. All detected processes are superimposed on the internal friction background that decreases in intensity as the temperature ∼+200 ${}^{{}^{\circ}}$C. Atomic–molecular treatment of the nature of each dissipative process is given.

Calculation of modulus defects ΔG for investigated PE has shown that β and $\beta_{k}$ processes have a relaxation mechanism of internal friction, and μ-process has a non-relaxation mechanism.

The analysis of the obtained dependencies shows the generality of the modulus defect change in the area of crystallinity degree of about 45%. This qualitative generality is determined by the fact that, independently of α, % for all dissipative processes detected on spectra (β, μ and $\beta_{k}$) there is an anomaly of the extreme form, which is expressed as a certain peak in the dependence of $\Delta G=f\left(\alpha ,\%\right)$.

Splitting $\beta_{k}$-dissipative loss peak on the spectrum of internal friction may be due to the formation of a number of structural–kinetic subsystems built from the same structural units, but with different structural organization in the amorphous–crystalline PE structure.

A methodology for the quantitative calculation of the temperature dependence of the shear modulus in a wide temperature range in the regime of a freely damped oscillatory process excited in the polymer sample under study taking into account the local temperature defects of the shear modulus caused by dissipative losses introduced by each dissipative process manifested on the spectrum of $\lambda =f\left(T\right)$.

## Figures and Tables

**Table 1:** Main physico-chemical characteristics according to manufacturers’ data sheets and specifications.

PE Grade	HDPE 277-73	HE3490-IM	CRP 100 Hostalen	Stavrolen PE4PP-25B
Somonomer type	ethylene	hexene	butene	butene
Molecular weight distribution (polydispersity)	Monomodal-	Bimodal-		Monomodal-
Density at 23 ${}^{{}^{\circ}}$C, g/cm${}^{3}$	0.957	0.962	0.960	0.952
Melt Flow Rate, g/10 min				
190 ${}^{{}^{\circ}}$C/21.6 kg	-	-	6.4	12.0–16.0
190 ${}^{{}^{\circ}}$C/5.0 kg	17.0–25.0	0.55	0.23	0.45–0.65
Shatter Point, ${}^{{}^{\circ}}$C, not higher	−50	-	-	−70
DSC Melting Point, ${}^{{}^{\circ}}$C	131	131	131	129
$\Delta H_{m}$, J/g	157.1	142.6	132.4	107.8
Degree of crystallinity, α, %	54	49	45	37

**Table 2:** Main physico-mechanical and physicochemical characteristics for dissipative loss process $\beta_{k}$.

PE Grade	HDPE 277-73	HE3490-IM	CRP 100 Hostalen	Stavrolen PE4PP-25B
Degree of crystallinity, α, %	54	49	45	37
$T_{\beta_{k\max}}$, ${}^{{}^{\circ}}$C	42	55	57	45
$\lambda_{\beta_{k\max}}$	0.310	0.440	0.379	0.387
$\nu_{\beta_{k\max}}$, Hz	1.79	2.29	1.94	1.96
$U_{\beta_{k\max}}$, KJ/mol	70.9	73.1	73.9	71.2
$\tau_{\beta_{k\max}}$, s	0.089	0.070	0.082	0.081
ΔT (at $\lambda =1/2\lambda_{\max}$)	95.7	93.2	90.5	92.4
Δτ (at $\lambda =1/2\lambda_{\max}$)	2.62	1.50	2.37	2.63
ΔG	0.82	0.94	0.90	0.91

**Table 3:** Experimental values of temperatures, frequencies and calculated values of modulus defects for all most pronounced peaks of dissipative losses detected on the spectrum (β, μ and $\beta_{k}$).

PE Grade		HDPE 277-73	HE3490-IM	CRP 100 Hostalen	Stavrolen PE4PP-25B
α, % (DSC)		54	49	45	37
*T*. ${}^{{}^{\circ}}$C	$\beta_{1}$	−136	−145	−149	−144
	$\beta_{2}$	−85	−87	−78	−87
	$\mu_{1}$	−85	−56	−52	−84
	$\mu_{2}$	−73	−43	−48	−77
	$\beta_{k1}$	−31	−37	−32	−42
	$\beta_{k2}$	103	111	102	84
ν. Hz	$\beta_{1}$	4.00	7.53	5.47	6.09
	$\beta_{2}$	2.99	4.81	3.71	4.24
	$\mu_{1}$	2.99	4.21	3.60	4.18
	$\mu_{2}$	3.17	4.60	4.00	4.47
	$\beta_{k1}$	2.71	4.47	3.62	3.94
	$\beta_{k2}$	1.16	1.13	1.14	1.16
ΔG	β	0.44	0.59	0.54	0.52
	μ	−0.12	−0.19	−0.23	−0.14
	$\beta_{k}$	0.82	0.94	0.90	0.91

## Data Availability

The data presented in this study are available on request from the corresponding author.
